# Why and how to investigate the role of protein phosphorylation in ZIP and ZnT zinc transporter activity and regulation

**DOI:** 10.1007/s00018-020-03473-3

**Published:** 2020-02-19

**Authors:** T. E. Thingholm, L. Rönnstrand, P. A. Rosenberg

**Affiliations:** 1 Department of Molecular Medicine, Cancer and Inflammation Research, University of Southern Denmark, J.B. Winsløws Vej 25, 3, 5000 Odense C, Denmark; 2 Division of Translational Cancer Research, Lund University, Medicon Village, Building 404, Scheelevägen 2, Lund, Sweden; 3 Lund Stem Cell Center, Lund University, Medicon Village, Building 404, Scheelevägen 2, Lund, Sweden; 4 Division of Oncology, Skåne University Hospital, Lund, Sweden; 5 Department of Neurology and F.M. Kirby Neurobiology Center, Boston Children’s Hospital, 300 Longwood Ave, Boston, MA 02115 USA; 6 Department of Neurology and Program in Neuroscience, Harvard Medical School, Boston, MA 02115 USA

**Keywords:** Zinc signaling, Protein phosphorylation, ZIP, ZnT

## Abstract

Zinc is required for the regulation of proliferation, metabolism, and cell signaling. It is an intracellular second messenger, and the cellular level of ionic, mobile zinc is strictly controlled by zinc transporters. In mammals, zinc homeostasis is primarily regulated by ZIP and ZnT zinc transporters. The importance of these transporters is underscored by the list of diseases resulting from changes in transporter expression and activity. However, despite numerous structural studies of the transporters revealing both zinc binding sites and motifs important for transporter function, the exact molecular mechanisms regulating ZIP and ZnT activities are still not clear. For example, protein phosphorylation was found to regulate ZIP7 activity resulting in the release of Zn^2+^ from intracellular stores leading to phosphorylation of tyrosine kinases and activation of signaling pathways. In addition, sequence analyses predict all 24 human zinc transporters to be phosphorylated suggesting that protein phosphorylation is important for regulation of transporter function. This review describes how zinc transporters are implicated in a number of important human diseases. It summarizes the current knowledge regarding ZIP and ZnT transporter structures and points to how protein phosphorylation seems to be important for the regulation of zinc transporter activity. The review addresses the need to investigate the role of protein phosphorylation in zinc transporter function and regulation, and argues for a pressing need to introduce quantitative phosphoproteomics to specifically target zinc transporters and proteins involved in zinc signaling. Finally, different quantitative phosphoproteomic strategies are suggested.

## Introduction

Zinc has been implicated as a factor in the development and progression of many pathological conditions such as cancer, inflammation, diabetes as well as in neurological and psychiatric diseases [1–14]. An estimated 3000 proteins interact with zinc, representing 10% of the genome, and zinc is a known regulator of gene expression through metal-responsive transcription factor-1 [15–19]. Furthermore, zinc has been identified as an intracellular second messenger involved in regulating various pathways, adding an extra dimension to its role in cellular regulation [20–22]. The basal level varies in different cell types. It ranges from tens to hundreds of pM free zinc, and it is strictly controlled as deviations from the normal cellular level may be cytotoxic [23, 24]. As zinc flux is primarily controlled by zinc transporters, we expect zinc-related dysfunction is not likely to result from dietary zinc deficiency or abundance alone, but rather from deviations in the function of proteins regulating zinc homeostasis.

The review gives a brief introduction to the role of zinc transporters in human diseases as well as evidence linking protein phosphorylation of zinc transporters to zinc signaling. The case is made that quantitative phosphoproteomics is an important approach to now understand more fully the role of phosphorylation in regulating zinc homeostasis. Finally, different quantitative phosphoproteomic strategies are suggested. In addition to zinc ions, ZIP and ZnT transporter are capable of transporting other cations such as iron, manganese, and cadmium. The mechanisms for transport of these ions and their link to different diseases is described elsewhere, and will not be discussed in this review as our focus will be on zinc transport [25–33].

## Zinc transporters

Four major zinc transporter families have been identified: (1) P-type ATPases; (2) RND (resistance, nodulation and division) multidrug efflux transporters; (3) the Zrt-, Irt-like protein (ZIP) family (Slc39A); and (4) the superfamily of cation diffusion facilitators (CDF) that includes the ZnT family of zinc transporters. The P-type ATPases are identified in bacteria and plants [34, 35], whereas the RND transporters only exist in a few Gram-negative bacteria [36]. In humans, the uptake of zinc is regulated by zinc transporters of the ZIP family (Slc39A) located in membranes such as the plasma membrane, the endoplasmic reticulum, and Golgi [37]. These transporter proteins import zinc from the extracellular environment or from organelles to increase the concentration of cytosolic zinc [37–39]. On the other hand, the mammalian CDF family and the Zn transporter (ZnT) family (Slc30A) mediates the export of zinc from the cytosol into organelles or out of the cell [37, 40]. ZnT transporters are found in membranes of intracellular organelles except for ZnT1 which is located in the plasma membrane [1, 41–43]. These two transporter families account for 14 human ZIP proteins and 10 human ZnT proteins and are responsible for controlling zinc homeostasis. In addition, zinc binding proteins such as metallothioneins assist in regulating the level of free zinc in the cell.

## Zinc transporters in diseases

Changes in the expression and activity of different zinc transporters have been directly linked to both systemic and central nervous system diseases, and to rare diseases such as acrodermatitis enteropathica (AE) [44] as well as lifestyle diseases and conditions affecting large numbers of people worldwide [45–58].

### Diabetes

ZnT8 is the zinc transporter best studied in diabetes. It is expressed in pancreatic beta cells and functions as target autoantigen in patients with type 1 diabetes [59]. A common W325R variant in the ZnT8 large C-terminal domain (CTD) has been associated with changed autoantibody specificity in type 1 diabetes as well as increased risk of developing type 2 diabetes [60]. ZIP14 and ZIP4 were also found to be involved in diabetes as altered zinc trafficking in *Zip14*^*−/−*^ mice resulted in a phenotype with defects in glucose homeostasis [45], and in the murine pancreatic beta cell line MIN6, overexpression of ZIP4 leads to increased granular zinc content and glucose-stimulated insulin secretion [50].

### Neurological and psychiatric diseases

In the nervous system, both transporters controlling zinc influx as well as zinc efflux play key roles in cellular regulation. The increase in cytosolic zinc mediated by ZIP12 leads to CREB phosphorylation and activation which is important for neuronal differentiation [61]. A mutation in the *SLC39A8* gene encoding the zinc and manganese transporter ZIP8 results in low levels of both Zn^2+^ and Mn^2+^ in the blood and increased levels in urine due to increased renal wasting in the affected patients [62]. The resulting autosomal-recessive disorder is characterized by intellectual disability, developmental delay, hypotonia, strabismus, cerebellar atrophy, and variable short stature [62]. ZnT3 has been identified as critical for transport of zinc into synaptic vesicles of a subset of glutamatergic neurons [63–65], and ZnT3 expression is reduced in patients with Alzheimer’s disease [66, 67] and Parkinson’s disease-related dementia [68]. Moreover, ZnT3 expression decreases with age suggesting a role in the prevention of aging-related cognitive loss [69]. Recently, Whitfield et al. proved a link between reduced ZnT3 protein level and depression in patients with dementia [14]. In a study in postmortem brain tissue, Rafalo-Ulinska et al*.* found a reduction in the ZnT3 protein level as well as a significant increase in the level of ZnT1, ZnT4, ZnT5 protein in the prefrontal cortex of subjects diagnosed with major depressive disorder (MDD) and in non-diagnosed suicide victims, relative to control subjects suggesting that zinc transporters are important in the pathophysiology of MDD and suicide [12]. ZnT3 is also inked to increased risk of febrile seizures [63, 70]. Changes in the level of ZnT transporers have also been identified in rats subjected to olfactory bulbectomy (OB), which is a model of depression [13].

### Cancer

Dysregulation of zinc homeostasis is critical in a variety of cancers. ZIP4 is found to be upregulated in several types of cancer cells [53, 54, 58]. ZIP6 also plays an important role in numerous cancers, particularly breast cancer [47, 49, 56, 57, 71]. Overexpression of ZnT2 is found to decrease invasive phenotypes of breast cancer cells [72]. In addition, reduced ZnT4 expression is observed in the progression from benign to invasive prostate cancer [73].

### Immune response

ZIP10 is important for B-cell survival and function and along with ZIP6 is involved in antigen presentation to T cells [46, 52]. Dendritic cells are important for immune response as they are involved in antigen presentation to T cells [74]. When dendritic cells are exposed to lipopolysaccharides, it results in the fragmentation of a ligand for toll-like receptor 4 (TLR4) from the outer membrane of Gram-negative bacteria. The TLR4–TRIF (Toll/Il-1 receptor domain-containing adapter protein-inducing interferon β) axis is activated leading to the downregulation of ZIP6 and ZIP10, and upregulation of ZnT1 and ZnT6 resulting in a significant drop in the intracellular zinc level [52]. ZIP8 is highly involved in inflammation [55], and was recently reported to be central to the development of osteoarthritis [51]. ZnT5 is required for the mast cell-mediated delayed-type allergic response by playing a role in Fc epsilonRI-induced translocation of protein kinase C (PKC) to the plasma membrane and in the nuclear translocation of nuclear factor kappaB [75].

## ZnT and ZIP structures

No three-dimensional structures of full-length human ZnT or ZIP proteins have been solved. Instead, most structural information on these transporters comes from sequence analysis and prediction studies as well as data on the crystal structures of prokaryotic homologs [76, 77]. The current knowledge on zinc transporter structure and function is described in detail in recent reviews [46, 69, 78].

### ZnT transporters

**Fig. 1:**
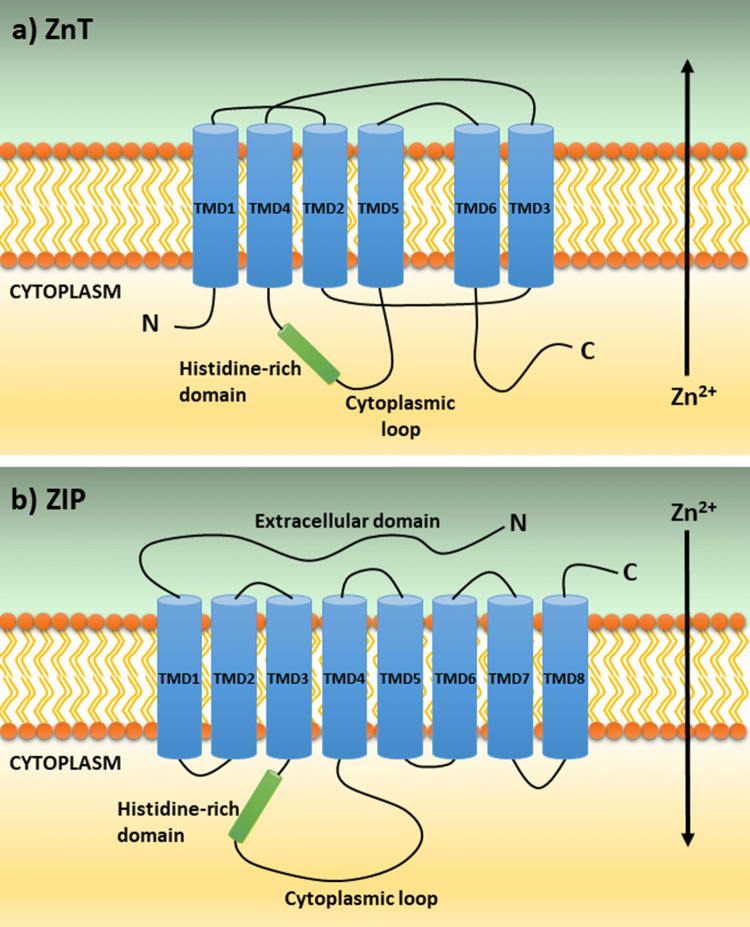
Predicted structures of ZnT and ZIP transporter proteins. **a** Predicted structure of members of the Zn transporter (ZnT) family (Slc30A) adapted from [79]. **b** Predicted structure of members of the Zrt-, Irt-like protein (ZIP) family (Slc39A). The long extracellular domain at the N-terminus is unique for members of the LIV-1 subfamily

Based on the three-dimensional structure of *Escherichia coli* homolog YiiP, the ZnT transporters have been predicted to contain six transmembrane domains (TMDs) with intracellular NH_2_ and COOH termini (Fig. 1a). The exception is ZnT5, which contains seven TMDs [78]. The literature presents different predictions for the location of the TMDs but overall, the ZnTs are described with a histidine/serine-rich domain in a cytosolic loop of varying lengths between TMD4 and TMD5, and a large CTD with an overall structural similarity with the copper metallochaperone Hah1, although the ZnT CTDs do not share sequence homology with Hah1 [77] **(**Fig. 1a).

Based on studies of YiiP, TMD1, TMD2, TMD4, and TMD5 are thought to form a compact four-helix structure in which four conserved hydrophilic coordination residues in TMD2 and TDM5 form a intramembranous zinc-binding site [69]. Mutations of these conserved residues have been shown to inhibit zinc transport activity [80]. Three of these coordination residues are conserved between bacteria and human, whereas the fourth site is a histidine residue in ZnT but alternates between a histidine and a aspartate residue in YiiP [81]. This enables ZnT, in contrast to YiiP, to discriminate between Zn^2+^ and the toxic ion Cd^2+^ [81]. Other studies point to the cytoplasmic domain as important for transporter activity as described for ZnT8 [60], as well as in ZnT10 where a L349P missense mutation in the CTD affects transporter activity, and a synonymous mutation (M250P) in the prokaryotic CDF protein, MamM, was found to be critical for the CTD fold illustrating the importance of the CTD [82]. The identification of a CDF superfamily lacking the CTD, however, points to additional strategies for zinc transport by ZnT transporters [43]. Furthermore, a di-proline motif in the luminal loop 2 conserved in ZnT5 and ZnT7 was recently found to be important for activation of the secretory and membrane-bound zinc-requiring enzyme, tissue-non-specific alkaline phosphatase (TNAP) [83].

### ZIP transporters

Based on sequence similarity, the ZIP family is divided into subfamily ZIP I, ZIP II, gufA, and LIV-1 (Liverpool-1) [84–86]. The ZIP1, ZIP2, and ZIP3 proteins belong to the ZIP II subfamily, ZIP9 to the ZIP I subfamily and ZIP11 to the gufA subfamily. The remaining nine ZIP proteins are part of the LIV-1 subfamily. These transporters are related to the estrogen-regulated gene, *LIV-1* and have primarily been identified in mammals [85]. The ZIP transporters contain eight putative transmembrane domains (TMDs) with extracellular NH_2_ and COOH termini (Fig. 1b) [38, 69, 78, 79, 87]. They all have a very short COOH terminus and a large cytoplasmic loop between TMD3 and TMD4 [46, 85, 88]. The cytoplasmic loop contains a histidine-rich motif with metal-binding properties [85]. In addition, members of the LIV-1 subfamily have a HEXXH motif in their TMD5 expected to function as a metal binding site and their sequences include a large NH_2_-terminal extracellular domain (ECD) not found in other ZIP subfamilies [85]. The ECD contains a highly conserved proline–alanine–leucine (PAL) motif, and for several of the LIV-1 subfamily members, these highly variable ECDs contain a high degree of histidine residues [69, 78, 85]. The expression of ZIP transporters at the cell surface is generally found to be upregulated upon dietary zinc deficiency (except for ZIP5) underscoring the tight connection between expression/function of the transporters and zinc signaling [89–91]. However, limited experimental data hamper our understanding of the regulation of zinc transport and zinc signaling in humans.

The ZIP4 protein is the best characterized of the ZIP transporters. ZIP4 is the ZIP transporter responsible for dietary zinc uptake as it is the exclusive transporter expressed on the apical surface of the intestinal epithelium [89]. Reduced zinc uptake caused by mutations in the *ZIP4* gene results in the rare autosomal recessive disorder acrodermatitis enteropathica (AE) [44]. Fifteen missense mutations in ZIP4 cause AE, of these, seven are found in the ECD emphasizing the central role of this domain [92, 93]. Recently, the first crystal structure of a mammalian ZIP4-ECD was reported [94]. The study revealed two independent subdomains; a helix-rich domain and a PAL motif containing domain connected by a short loop and stabilized by four disulfide bonds. The study showed how two ECDs form a dimer centered at the PAL motif. As some of the AE-causing mutations eliminate the first and the fourth disulfide bond and downregulate ZIP4 glycosylation in mice, the two disulfide bonds are found to be critical for ZIP4 folding [94].

ZIP4 is found to be important for zinc transport activity, transporter processing and trafficking [94–96]. During zinc deficiency, the ZIP4-ECD is cleaved to form a ~ 35 kDa ZIP4 peptide. This peptide accumulates as a peripheral membrane protein, whereas the remaining ~ 37-kDa ZIP4 COOH-terminal processed peptide accumulates as an integral membrane protein [96]. Two AE mutations are found to block ECD cleavage and other mutations have been shown to diminish cleavage suggesting that proteolytic processing of ECD is important for ZIP4 function in regulating zinc homeostasis [96]. ECD cleavage has been found to occur in both ZIP4, ZIP6, and ZIP10 [97].

Dempski and co-workers investigated the function of the large human ZIP4 (hZIP4) cytoplasmic loop between TMD3 and TMD4 (M3M4) [98]. Combining site-directed mutagenesis, metal binding affinity assays, and X-ray absorption spectroscopy demonstrated how two Zn^2+^ ions bind sequentially to two different sites in the cytosolic loop due to different zinc affinities [98]. First, one Zn^2+^ ion binds to a CysHis3 site with a nanomolar binding affinity. Then a second Zn^2+^ ion binds to a His4 site with a weaker affinity, suggesting a form of zinc sensing role of the M3M4 domain, perhaps involved in the regulation of the ZIP4 level at the plasma membrane according to the need for cytosolic zinc [98].

Studies of hZIP4 and the mouse homologue (mZIP4) have shown how the cytosolic concentration of zinc ions regulate ZIP4 surface expression [88]. The level of murine ZIP4 mRNA in enterocytes and yolk sac is increased upon zinc limitation and suppressed upon zinc repletion [89, 99], and mZIP4 protein expression at the plasma membrane is reduced when zinc-deficient mice are fed a zinc-replete diet [89]. High-cytosolic Zn^2+^ concentrations were found to reduce mZIP4 expression at the plasma membrane through Zn^2+^-dependent endocytosis [100]. Kim et al*.* demonstrated that both mZIP4 and hZIP4 protein accumulate at the plasma membrane during zinc deficiency and undergo endocytosis when cells are exposed to low zinc concentrations (~ 1 µM Zn) leading to reduced zinc uptake through ZIP4 [100]. Furthermore, Mao and co-workers showed how a histidine-rich cluster in the cytoplasmic loop mediates ubiquitination and proteasomal degradation of hZIP4 at higher concentrations of zinc to protect against zinc cytotoxity [101].

It is clear that the regulation of cellular zinc is both stringent and complex and that various strategies are used to control the level and activity of ZIP4, and other zinc transporters as well [102].

## General overview of protein phosphorylation

**Fig. 2:**
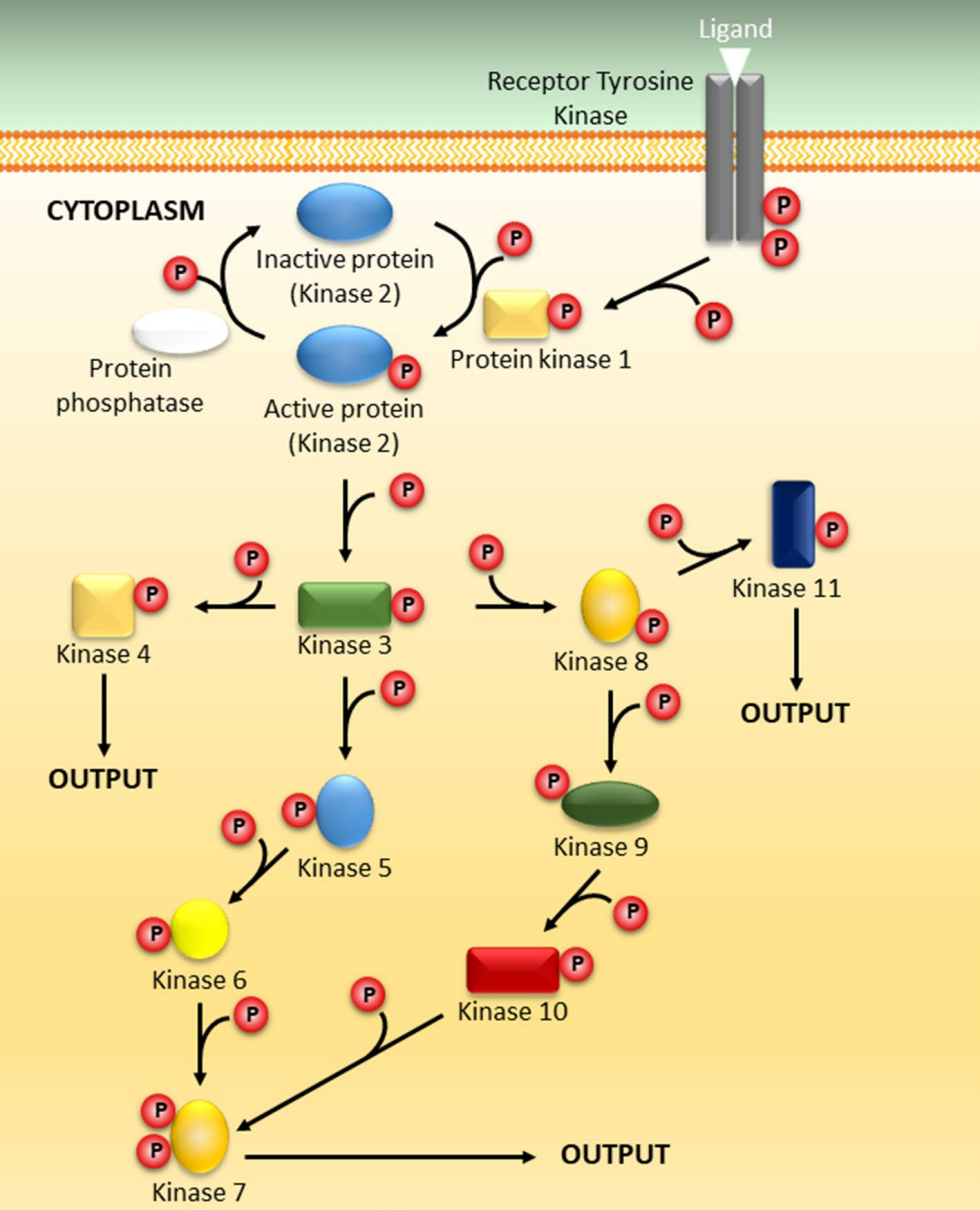
Signal transduction pathway. The figure illustrates the principle of a protein phosphorylation cascade. Protein phosphorylation of target proteins is catalysed by protein kinases, whereas dephosphorylation is catalysed by protein phosphatases. As an example, binding of a ligand to a receptor tyrosine kinase results in dimerization and autophosphorylation of the receptor. This leads to the phosphorylation and activation of kinase 2 initiating a phosphorylation cascade affecting several protein kinases. Through signal transduction pathways, the cell can propagate and enhance the cellular output. The cellular output depends on the specific pathway. Red P circle indicates protein phosphorylation. Double red P circles indicate protein phosphorylation at multiple sites. White figure—protein phosphatase. Coloured figures—different protein kinases

Protein phosphorylation is another post-translational modification (PTM) involved in zinc transport and signaling. It is a transient PTM that enables the cell to change the conformation, activity, and interaction of target proteins within a very short timeframe. It is a reversible modification, and a complex interplay between specific protein kinases and protein phosphatases keeps a strict temporal and spatial control of the phosphorylation and dephosphorylation of target proteins at specific sites. This enables the cell to quickly transduce extracellular signals into intracellular signals through signal transduction pathways in which a large range of target proteins are affected (Fig. 2). Protein phosphorylation is one of the most widespread regulatory mechanisms in nature, acting as an important modulator of intracellular biological processes such as proliferation, differentiation, cell survival, transcription, and translation [103–107]. Genomic sequencing indicates that 2–3% of all eukaryotic genes are likely to code for protein kinases [105], and at present, protein phosphorylation is the most studied and best understood post-translational modification [108, 109].

## Protein phosphorylation is linked to zinc transporters and zinc signaling

### Zinc and protein tyrosine phosphatases

A strong collaboration between zinc signaling and protein phosphorylation is clear from the actions of zinc as a second messenger in different cell types leading to the activation of various phosphorylation cascades, and the inhibition of protein tyrosine phosphatase (PTP) activity [20, 21]. PTPs play central roles in cellular function as key regulators of protein phosphorylation, directly affecting the activity of a great number of phosphoproteins and tyrosine kinase pathways [110–114]. PTPs contain a substrate binding site (in the P-loop) binding the phosphate ester group of the substrate and an active site (in the protein loop known as the WPD-loop due to the conserved tryptophan–proline–aspartic acid sequence). The WPD-loop exists in an open conformation in which the conserved aspartic acid is positioned 8–10 Å from the active site [115]. Substrate binding results in a conformational change in the WPD-loop leading to a closure of the loop placing it over the active site and bringing the conserved aspartic acid close to the bound substrate [115, 116]. Upon binding of a substrate, the nucleophilic Cys215 thiol in the P-loop attacks the substrate phosphate ester group. The leaving group is protonated by Asp181 in the WPD-loop forming a phospho-enzyme intermediate and releasing peptidyl tyrosine [115, 116]. Subsequent hydrolysis of the phospho-enzyme intermediate releases inorganic phosphate and regenerates the PTP enzyme with the WPD-loop in the open conformation [117, 118].

PTPs are regulated by protein phosphorylation, redox signaling, dimerization, and proteolysis. We suspect that zinc may inhibit PTP activity by affecting these processes directly or using other mechanisms. The PTP regulating the insulin and leptin signaling pathway, PTP1B (PTPN1) is one of the most extensively studied PTPs. Bellomo et al*.* optimised an assay for investigating zinc inhibition of PTPs using PTP1B [118]. They found the binding of zinc to PTP1B to require activation in the presence of a substrate as it only binds to the catalytic pocket of the closed PTP1B phospho-intermediate [118]. The analysis of three PTP1B 3D structures (PDB id: 2CM2, 3I80, and 1A5Y) revealed putative zinc binding sites and supported the hypothesis of inhibition only of the phospho-intermediate. Furthermore, they showed how PTP1B inhibition can also result from an alternative mechanism possibly initiated by the binding of zinc to the PTP1B surface. As PTP1B and the zinc transporter ZIP7 are both localized on the ER membrane, zinc influx through ZIP7 may play an important role in the regulation of PTP1B activity [118].

### Zinc and receptor tyrosine kinases

Zinc is found to induce phosphorylation of a variety of receptor tyrosine kinases including the insulin receptor (IR), IGF-1R, and EGFR resulting in the activation of the PI3K/Akt and Erk signaling cascades [119, 120]. This affects insulin-stimulated glucose metabolism and cell survival. Zinc also initiates phosphorylation and activation of Erk1/2 in neurons leading to neurotoxicity [121–124]. In 2002, Du et al. showed that a brief exposure to methylisothiazolinone, a widely used industrial and household biocide, resulted in a zinc-dependent activation of Erk1/2 via a 12-lipoxygenase-mediated pathway leading to neuronal death in neurons in culture [122, 123]. Later, Ho and co-workers found zinc accumulation resulting from oxidative stress to selectively inhibit Erk1/2-directed phosphatases either by increased degradation or reduced enzyme activity leading to increased kinase activity in the murine hippocampal neuronal cell line, HT22 cells, and immature neurons [121].

The neurotrophin receptor, TrkB, is required for neuronal survival and differentiation as well as synaptic structure, function, and plasticity [125, 126]. As a consequence, dysregulation of TrkB activity and signaling is involved in various neurological and psychiatric disorders [127–129]. TrkB is a receptor tyrosine kinase activated by the binding of neurotrophins leading to dimerization, increased kinase activity, and autophosphorylation of the receptor resulting in the activation of downstream phosphorylation cascades [130]. Zinc, however, is found to activate TrkB in a neurotrophin-independent manner [131, 132]. Activation results from increased activity of the Src family of protein tyrosine kinases. The TrkB activation by zinc has been shown to potentiate the hippocampal mossy fiber-CA3 pyramid synapse [132]. Also, Zn^2+^ induces the activation of Trk signaling pathways leading to multi-phosphorylation of the striatal-enriched phosphatase (STEP) as well as phosphorylation and activation of Erk2, a STEP substrate involved in Zn^2+^-dependent neurotoxicity [133].

### Zinc and NFκB

NFκB (NF-kappa B, nuclear factor kappa-light-chain-enhancer of activated B cells) is a major transcription factor also regulating immune responses to infection, stress, free radicals etc. NFκB is also important for synaptic signaling is NFκB [134]. In unstimulated cells, NFκB is located in the cytosol inactivated by the binding to inhibitory protein IκB (inhibitory proteins of NFκB). As a response to infection, the binding of pro-inflammatory cytokines stimulates the phosphorylation of IκB proteins by specific kinases such as IκB kinase (IKK). This leads to the ubiquitination and degradation of IκB. NFκB is subsequently released and free to translocate to the nucleus to stimulate the transcription of specific target genes by binding to their promoter regions [135–137]. The research group of Sarkar found zinc to activate NFκB in HUT-78 cells [138]. They also found zinc to be required for gene expression of both interleukin-2 (IL-2) and interleukin-2 receptor alpha and beta (IL-R2α and IL-R2β) which was also seen in vivo [139, 140]. Subsequently they showed that the upregulation of NFκB activity was a result of zinc stimulating IκB phosphorylation [141]. Increased protein tyrosine kinase (PTK) activity and reduced PTP activity caused by increased oxidative stress during aging was found to result in the phosphorylation of NFκB, NIK/IKK, and MAPKs (Erk, p38, and JNK) leading to NFκB activation [142, 143]. As a general point, zinc appears to play a key role in the regulation of PTK/PTP balance, by the activation of phosphorylation as a result of inhibition of PTPs or by direct activation of kinases.

### Zinc signaling in cancer cells

Zinc transporters have also been linked to protein phosphorylation and signaling in cancer cells. In MDA-MB-468 breast cancer cells and PC-3 prostate cancer cells transfected with ZIP9 (PC3-ZIP9) cells, ZIP9 was found to act as a specific Gi-coupled membrane androgen receptor (mAR) binding testosterone and initiating MAPK and zinc signaling leading to apoptosis [144, 145].

In the estrogen receptor-positive human breast cancer cell line MCF-7, the ability of cells to spread to the lymph nodes was found to be a result of ZIP6-mediated zinc influx [71]. Signal transducer and activator of transcription 3 (STAT3) and oestrogen induce transcription of ZIP6. Upon transcription, ZIP6 is proteolytically cleaved at the N-terminus to translocate to the plasma membrane where it mediates Zn^2+^ influx. The increase in cellular zinc then results in the phosphorylation and inactivation of glycogen synthase kinase-3beta (GSK-3β) possibly through insulin-mimetic actions of zinc or indirectly through activation of Akt [71, 146, 147]. The inhibition of GSK-3β prevents it from phosphorylating transcription factor Snail, which is then retained in the nucleus acting as a transcriptional repressor of the E-cadherin gene, *CDH1* (epithelial cadherin), CDH1, causing cell rounding, and detachment [71].

In 2012, it was shown in MCF-7 cells that the activity of human endoplasmic reticulum zinc channel hZIP7 was regulated by protein phosphorylation at Ser275 and Ser276 upon zinc stimulation [20]. Phosphorylation of hZIP7 was linked to the release of Zn^2+^ ions from intracellular stores which further led to the phosphorylation of tyrosine kinases such as Erk1/2 and Akt, and the subsequent activation of signaling pathways within the cell [20, 148]. Two additional phosphorylation sites at S294 and T294 have also been found to be important for maximal hZIP7 activity [113].

Besides being important for the regulation of cell signaling as mentioned above, protein phosphorylation is also likely to be central to the regulation of zinc transporter activity and regulation as in silico analysis predict all human ZnT and ZIP transporter proteins to be phosphorylated (Table 1 and Table 2). This underscores the importance of characterizing protein phosphorylation in zinc transporter proteins.

**Table 1:** Phosphorylation sites in human ZnT transporter proteins

Zinc transporter	#P-sites (predicted)	#P-sites (published)	P-sites (published)	References
ZnT1	39	15	S29, S167, S172, S173, T196, S426, S429, T439, T449, T462, S466, S468, S473, S505, S506	[149–156]
ZnT2	22	2	T239, S247	
ZnT3	34	3	S32, S38, T66, S311	[157]
ZnT4	40	4	S12, S75, S313, S412	
ZnT5	59	12	Y5, T30, Y32, T39, T88, S364, S378, T382, Y385, Y757, T762, Y763	[153, 158]
ZnT6	38	6	S122, T376, S381, S382, S388, T391,	[152, 159, 160]
ZnT7	29	2	Y11, S31	
ZnT8	25	2	T7, Y8,	
ZnT9	53	3	T222, S230, S355	
ZnT10	36	7	S187, S189, T192, S197, S402, S469, Y479	[161]

Number of predicted phosphorylation sites (#P-sites) in human ZnT transport proteins according to the NetPhos 3.1 Server is listed along with phosphorylation sites that have been experimentally identified and published according to UniProt and PhosphoSitePlus [162, 163]. Phosphorylation sites listed in the NetPhos 3.1 Server with a prediction score above 0.5 are included. References listed in the table only account for research articles. References for phosphorylation sites found on curated info pages are not listed in the table.

**Table 2:** Phosphorylation sites in human ZIP transporter proteins

Zinc transporter	#P-sites (predicted)	#P-sites (published)	P-sites (published)	References
ZIP1	14	0		
ZIP2	23	0		
ZIP3	26	3	S125, S129, Y147	[150, 159, 164]
ZIP4	57	2	T137, S490	[165, 166]
ZIP5	4	1	S336	[167]
ZIP6	34	11	S471, Y473, S475, S478, T479, T486, T490, Y493, Y528, Y531, S583	[152, 160, 168–172]
ZIP7	8	4	S275, S276, S293, T294	[113, 154, 173]
ZIP8	1	4	S275, S278, S288, T424	[172–174]
ZIP9	1	0		
ZIP10	20	12	S323, T536, S539, T540, S546, T553, S556, S570, S573, T583, S591, Y596	[149, 152, 155, 158, 159, 174–179]
ZIP11	4	2	S125, S153	[152, 153]
ZIP12	5	0		
ZIP13	3	3	S39, T42, T44	
ZIP14	9	5	S256, Y258, S260, S309, S311	[167, 169, 180, 181]

Number of predicted phosphorylation sites (#P-sites) in human ZIP transport proteins according to the NetPhos 3.1 Server is listed along with phosphorylation sites that have been experimentally identified and published according to UniProt and PhosphoSitePlus [162, 163]. Phosphorylation sites listed in the NetPhos 3.1 Server with a prediction score above 0.5 are included. References listed in the table only account for research articles. References for phosphorylation sites found on curated info pages are not listed in the table.

## Mass spectrometry and quantitative phosphoproteomics for studying zinc signaling

Despite the ubiquitous role of protein phosphorylation, phosphoproteins have low abundance within the cell due to the transient nature of phosphorylation. In addition, phosphate groups are easily lost during sample handling. These problems make the identification and characterization of phosphorylated proteins challenging, and for years, the study of protein phosphorylation was hampered by the lack of efficient and specific methods. Traditional molecular approaches frequently focus on the analysis of a single phosphoprotein and entire signaling pathways are studied protein by protein. One often has to work from the assumption or prior knowledge that specific proteins are phosphorylated and investigate this using approaches such as cloning, 1D or 2D gel electrophoresis combined with Pro-Q Diamond staining [182–190], radioactive labelling using ^32^P or ^33^P [182, 183], or directed antibody-based strategies limited by their low specificity and high cost. Furthermore, detection provides no information of the specific sites of phosphorylation unless antibodies have been generated against specific phosphorylation sites.

Mass spectrometry (MS) is a powerful tool for discovery-based analysis of proteins and phosphoproteins where no prior knowledge is required. In MS, the protein or peptide samples are ionized, separated according to their mass-to-charge ratios before the ions are registered by a detector. The measurement of the mass-to-charge ratio of a sample molecule provides exact details on the mass, sequence and PTMs which in turn can give useful information about the structure, interaction and regulation of the particular molecule. The analysis of protein phosphorylation by MS, termed phosphoproteomics, requires selective phospho-specific enrichment methods in combination with MS strategies. Phosphorylation-specific antibodies have been used at both the protein and peptide levels. However, the poor specificity and sensitivity of anti-phosphoserine and anti-phosphothreonine antibodies have limited their use leaving the more specific anti-phosphotyrosine antibodies as the preferred method for antibody-based enrichment [191–198]. In nature, however, the vast majority of protein phosphorylation is on serine and threonine residues [199].

For many years, the primary method for phosphopeptide enrichment prior to MS analysis was immobilized metal ion affinity chromatography (IMAC) using the affinity of negatively charged phosphate groups on proteins and peptides towards positively charged metal ions (Fe^3+^, Al^3+^, Ga^3+^, or Co^2+^) chelated to nitriloacetic acid (NTA) or iminodiacetic acid (IDA)-coated beads [125, 200–208]. IMAC, however, has a limited selectivity when used on complex samples, and requires an O-methylesterification step for improved selectivity adding more complexity to the sample [209]. Titanium dioxide (TiO_2_) was found to have affinity for phosphate ions in aqueous solutions [210–216]. This initiated great progress in the development of new and highly sensitive strategies for phospho-specific enrichment before MS analysis [214, 217–220], and TiO_2_ chromatography quickly replaced all other strategies as the method of choice for large-scale phosphoproteomic studies [176, 179, 214, 217–228]. The reason for the popularity of the method is the high selectivity toward phosphopeptides but also due to the setup being simple and fast, and that TiO_2_ beads, unlike IMAC, are extremely tolerant toward most buffers and salts used in cell biology laboratories [229]. Later, the strengths of both IMAC and TiO_2_ chromatography were combined in the SIMAC method (sequential elution from IMAC) improving not only the number of phosphorylation sites detected from complex samples, but also significantly increasing the detection of multiply phosphorylated peptides from low amounts of material [230, 231]. These strategies allow for large-scale phosphoproteomic screening of various cell lines and tissues [214, 217–220, 230]. Indeed, all but one phosphorylation site identified in human ZnT and ZIP transporters have been identified in large-scale phosphoproteomic studies (Tables 1, 2).

The use of phosphoproteomic strategies can be very powerful. By combining these methods with quantitation strategies and highly efficient MS technology the comparison of proteins and PTMs in different cells, disease stages, treatments etc. is possible. Common quantitative strategies include the introduction of different isotopic labels to proteins or peptides from samples to be compared. Subsequent MS analysis then reveals differences in the abundance of labelled species originating from the different samples. Labelling can be introduced at the protein level using metabolic labelling such as ^14/15^ N-labeling or stable isotope labelling by amino acids in cell culture (SILAC) where different isotopic tags are added to the media and incorporated into proteins during cell culturing [232]. Chemical modification is introduced either during protein digestion (O^18^-labelling) or at peptide level using isobaric peptide tags for relative and absolute quantification (iTRAQ), tandem mass tags (TMT) or dimethyl labelling introduced during sample preparation [233–238]. In addition, MS offers the use of label-free strategies where quantitation is performed by the comparison of liquid chromatography (LC)-MS or MS/MS data obtained in sequential experiments [239, 240]. The different strategies within phosphoproteomics and quantitative phosphoproteomics are described in various reviews [241–244].

## Conclusions and future perspectives

Despite the power of these strategies, phosphoproteomic approaches have barely been used in the study of zinc homeostasis and zinc signaling. Except for one phosphorylation site, all sites identified in the ZnT and ZIP transporters were discovered in large-scale screenings to characterize phosphorylation profiles of different cell types or conditions or during the development of new MS strategies to identify even more phosphorylation sites [149–161, 164–181]. None of the studies were designed to investigate zinc homeostasis or zinc signaling. Instead the ZnT and ZIP protein phosphorylation sites were all identified by chance. The time is ripe for using these sensitive strategies to specifically target zinc transporters and proteins involved in zinc signaling. Quantitative phosphoproteomic approaches have already proven their potential in biological studies [245–249].

The low-abundance and highly hydrophobic nature of membrane proteins adds additional challenge to the identification of phosphorylation sites in zinc transporter proteins which could explain why the sites identified primarily come from large-scale studies using large amounts of starting material. Over-expression of specific zinc transporters combined with membrane enrichment strategies would improve identification in discovery-based studies. The identification of novel phosphorylation sites in ZnT and ZIP proteins will enable the investigation of possible cytosolic binding partners in pull-down experiments using phospho-specific antibodies directed against these novel sites.

In addition, these approaches will be useful for understanding zinc physiology from studies of over-expressing or knocking out zinc transporters, time and dose dependent zinc stimulation, or studying specific diseases linked to aberrant zinc homeostasis. Combining such data with molecular methodology will lead to a deeper understanding of the molecular mechanisms regulating zinc homeostasis and shed light on the signaling pathways affected by zinc stimulation.
